# Mechanical Properties and Microstructure of Polypropylene–Glass-Fiber-Reinforced Desert Sand Concrete

**DOI:** 10.3390/polym15244675

**Published:** 2023-12-11

**Authors:** Lina Hou, Baojun Wen, Wei Huang, Xue Zhang, Xinyu Zhang

**Affiliations:** 1 College of Architecture Engineering, Xi’an Technological University, Xi’an 710021, China; zhang231204@163.com (X.Z.); zxy0103242022@163.com (X.Z.); 2 Xi’an Survey, Design and Research Institute Co., Ltd. of CREC, China Railway Liu Yuan, Xi’an 710021, China; wenbaojun@163.com; 3 College of Civil Engineering, Xi’an University of Architecture and Technology, Xi’an 710055, China; qqhuangwei2005@126.com

**Keywords:** desert sand concrete, hybrid fiber, mechanical properties, pore structure, microstructure

## Abstract

In order to improve the performance of desert sand concrete, polypropylene fiber (PF) and glass fiber (GF) were used to prepare desert sand concrete (DSC) with different fiber and volume content, and the basic mechanical properties, such as cube compressive, tensile and flexural strengths, were tested and studied. Based on the mercury injection method (MIP) and scanning electron microscopy (SEM), the evolution of pore structure and interface structure was analyzed. The mechanism of fiber toughening was revealed at the microscopic level. The results show that the slump of DSC decreases with the increase in fiber content. The slump of glass-fiber-reinforced DSC (GFRDSC) is smaller than that of polypropylene-fiber-reinforced DSC (PFRDSC). The strength enhancement of DSC by fibers is in the order of flexural strength > split tensile strength > compressive strength. The flexural strength of hybrid-fiber-reinforced DSC (HyFRDSC) (0.1% PF + 0.1% GF) is increased by 40.7%. Meanwhile, fibers can improve the toughness of DSC. The MIP results show that the porosity of HyFRDSC decreased by 50.01%, and the addition of fiber can effectively refine the large pore size. The SEM results show that the incorporation of PF and GF causes the formation of a uniform and dense structure between the fibers, cement and aggregate. The two can give full play to the crack-resisting and toughening effect in different loading stages, thus improving the macromechanical properties of DSC.

## 1. Introduction

Accelerated global infrastructure construction unprecedentedly increases the demand for concrete and sand. However, meeting engineering and construction needs is challenging given the limited natural sand resources [1]. The long-distance transport of natural sands raises construction costs and greenhouse gas emissions, leading to serious environmental problems and river sand resource consumption [2,3,4]. For this reason, alternatives to sand resources are urgently required [5,6]. Desert sand, a weathering product of rock, has a small fineness modulus [7] and huge deposits in vast desert areas worldwide. Partially replacing natural sand with desert sand has far-reaching significance in reducing construction costs, protecting the ecological environment and satisfying engineering application standards and conditions.

Desert sand can refine microstructure and fill pores, improving cement slurry density [8,9,10]. Therefore, the mechanical properties of desert sand concrete (DSC) are very close to those of ordinary concrete, and the desert sand can be used as a fine aggregate in civil engineering concrete to meet the general engineering requirements. However, due to its low fineness modulus, high water requirement and easy cracking, shrinkage and bleeding, the application of desert sand resources is limited [11,12,13,14].

Fiber-reinforced concrete technology has been widely used, and the bonding effect between fiber and the concrete matrix can enhance the tensile properties of concrete and reduce the occurrence of crack generation and expansion in the matrix, improving the mechanical properties of concrete. Fiber-reinforced concrete can be widely used in construction engineering, water conservancy engineering, road and bridge engineering and tunnel and military engineering [15,16]. Hybrid-fiber-reinforced concrete is a high-performance composite construction material formed by mixing various fibers into concrete according to a certain ratio. Conventional fiber combinations include macrofibers (steel fiber, glass fiber (GF), carbon fiber, aramid fiber (AF), etc.) and microfibers (polyvinyl alcohol fiber (PVAF), polypropylene fiber (PF), polyester fiber, polyacrylonitrile fiber, etc.) [17].

Studies have shown that hybrid-fiber-reinforced concrete has better mechanical properties than single-mixed-fiber-reinforced concrete. The influences on the mechanical properties of mixed-fiber-reinforced concrete come mainly from the type of fiber and the mixing ratio. For example, the compressive strength, splitting tensile strength and bending strength of concrete mixed with steel fibers and PFs were superior to those of concrete only mixed with steel fibers; the maximum increase in compressive strength could reach 50.1% [18,19], and the optimum fiber content was 1.5% steel fibers and 1.5% PFs [19]. Blending basalt fibers, PVAFs and steel fibers could achieve the optimal synergistic performance, and the compressive and flexural strength of concrete was significantly increased [20]. The favorable blending effect of steel and carbon fibers largely also improved the compressive strength and toughness of concrete [21]. Steel fibers when blended with polyolefin fibers, PVAFs, GFs, polyester fibers and basalt fibers, respectively, increased the tensile strength of UHPC more than single steel fibers [22]. The best mechanical properties of concrete were obtained with 0.8% basalt fibers and 0.25% steel fibers [23]. The flexural and tensile strength of PVA–steel-fiber-reinforced concrete was largely increased with the elevating mix proportion of steel fibers [24]. The compressive strength and elastic modulus of PVAF/PPF-reinforced concrete dropped with the rising volume ratio of PFs [25]. The mixture of 0.15% basalt fiber and 0.033% PFs had the best synergistic effect [26]. In addition, the shape of steel fibers dramatically enhanced the flexural strength of steel-fiber–PF-reinforced concrete [27]. The length-to-diameter ratios of PFs and AFs greatly influenced the early mechanical properties of concrete [28].

PFs and GFs have shown a favorable performance in improving the properties of concrete and are extensively applied in engineering projects such as road pavements, bridges and harbors [29]. PFs are favored in concrete reinforcement due to their low cost, easy processing, high strength, low density and good corrosion resistance. Adding PF to concrete can considerably improve the economic benefits and sustainability [30,31]. PF greatly enhances the mechanical properties and crack resistance of concrete [32] in the case of 0.5% fiber content. The splitting strength and flexural strength of concrete were increased by 12.01% and 17.15%, respectively [33]. Additionally, 0.1–0.3% PF can reduce the plastic shrinkage of concrete by 12–25%, and the tension–compression ratio can be increased by 46% [34,35,36]. 

Alkali-resistant GF is a new fiber material characterized by high ductility, strong temperature resistance, noncombustibility, favorable corrosion resistance, large tensile strength and good thermal, acoustic and electrical insulation. GF can cross over and inhibit the development of macroscopic cracks [37], increasing the compressive strength, flexural strength and tensile strength of concrete by 15%, 44.6% [38] and 10–17%; in addition, the deformability and energy absorption capacity were also intensified [39]. In view of the excellent hybrid effect of different fibers among blended groups, the combination of GF with a higher modulus of elasticity and PF with a lower modulus of elasticity can achieve complementary advantages to improve the cracking resistance and reinforcement properties of the materials. It was reported that when PF and GF dosages were less than 0.45%, the concrete properties were highly improved; at 1.5% hybrid-fiber content, the 28-day compressive and flexural strengths were increased by 9.35% and 20.36%, respectively [40,41].

Fiber-reinforced concrete technology has been applied in DSC. Some researchers experimentally found that mixing steel fibers could substantially enhance the compressive strength and splitting tensile strength of DSC, and the enhancement of the latter strength was relatively obvious [42,43,44]. Steel fibers account for the most commonly used option in the current literature, yet they have the disadvantages of self-weighting and easy corrosion [45]. Additionally, existing research mainly focuses on single-fiber-doped DSC; investigation into HyRDSC is still a research gap; mixed PF and GF reinforced DSC especially have not been reported.

In this paper, to further enhance DSC performance, HyRDSC with various fibers and volume admixtures was prepared using PFs and GFs, and its basic mechanical properties (compressive, flexural and splitting tensile strength) were experimentally investigated. In addition, the toughening mechanism of these fibers was further explored at the microscopic level based on mercury injection capillary pressure (MIP) tests and scanning electron microscopy (SEM). The current findings are significant for environmental protection, shortage alleviation of sand and gravel resources, achievement of sustainable development strategy and carbon emission reduction.

## 2. Materials and Methods

### 2.1. Raw Materials

**Figure 1:**
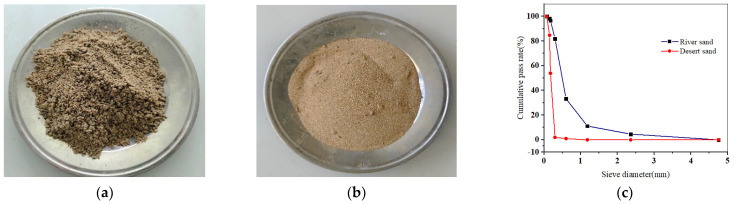
Fine aggregates and sieving curve: (**a**) river sand; (**b**) desert sand; (**c**) grading curve.

**Figure 2:**
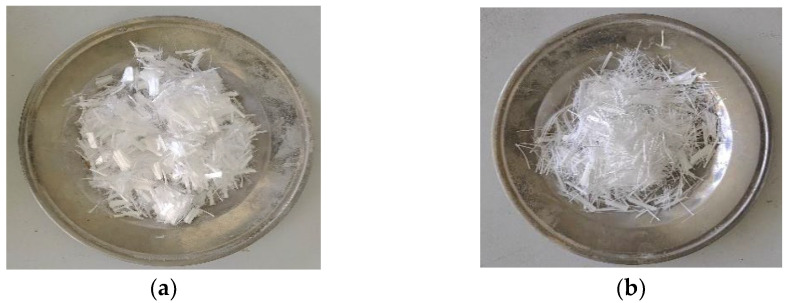
Fibers: (**a**) PFs; (**b**) GFs.

Grade PO·42.5 cement produced by Conch Cement Co., Ltd. (Xianyang, China) was used. The cement parameters are listed in Table 1, conforming to the provisions of GB/T 175-2020 [46]. The gravel coarse aggregates in this study have a maximum particle size of 20 mm. The sand for fine aggregate was collected from the rivers around Xi’an, and the desert sand was obtained from the Mu Us Desert in Yulin City; both are shown in Figure 1. The physical parameters and grading curve of the fine aggregates are presented in Table 2 and Figure 1 (GBT 14684-2011) [47]. Grade I fly ash from Henan Borun Co., Ltd. (Anyang, China) was selected, and the corresponding parameters are given in Table 3. PFs and GFs and their performance parameters are shown in Figure 2 and Table 4, respectively. A naphthalene-type superplasticizer with a water-reducing rate of 16% was employed. Tap water in Xi’an was utilized for concrete mixing.

### 2.2. Mix Proportions and Specimen Preparation

The concrete strength grade is C40. Based on extensive data [34,38,40,41] and repeated tests, the desert sand replacement ratio was set at 30%, and its benchmark is shown in Table 5 according to JGJ 55-2011 [48]. The selected mix ratio and the effects of fiber type and content on the compressive, flexural and splitting tensile strengths of DSC were studied. Twelve groups of specimens were designed, as shown in Table 6. According to GB/T50008-2019 [49], the specimens for testing compressive and tensile strengths were 100 mm^3^, and those for flexural strength measured 100 mm × 100 mm × 400 mm. Three test blocks without visible defects were selected for each group.

A dry-mixing method was applied to concrete to ensure fiber dispersion. Firstly, coarse aggregates, fine aggregates, cement and fly ash were added successively. The fibers were added after dry-mixing for 30 s, followed by the addition of water and a water-reducing agent after mixing for another 90 s. Secondly, the mixture was stirred for 1 min. Finally, the concrete was loaded into a mold, and the blocks were demolded after 24 h. The tests were carried out after standard curing for 28 d.

### 2.3. Test Methods

**Figure 3:**
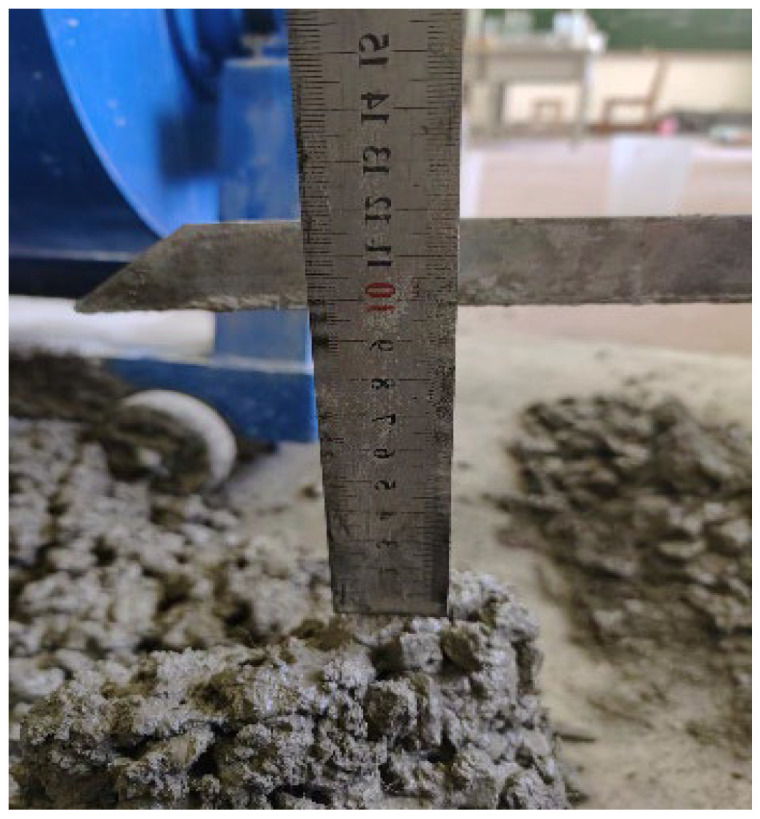
Slump test.

**Figure 4:**
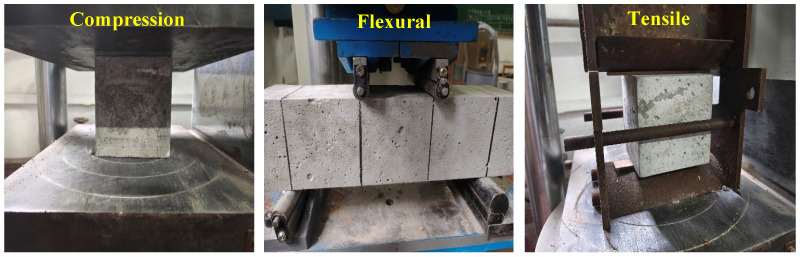
Mechanical test.

**Figure 5:**
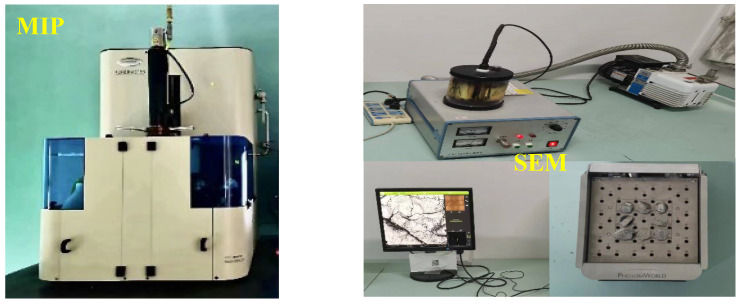
Pore structure and microstructure tests.

The effects of PFs and GFs on the mechanical properties and microstructure of DSC were investigated. The experimental methods are depicted in Figure 3, Figure 4 and Figure 5, including slump, mechanical, pore structure and microstructural tests.

The workability of fresh DSC was assessed through slump cone tests according to GB/T 50080-2016 [50]. As illustrated in Figure 3, the slump is measured in mm and is as precise as 1 mm.

The loading scheme and system for the macromechanical property tests were designed referring to GB/T 50081-2019 [49]. All tests were conducted on a TSY-2000 electrohydraulic pressure testing machine purchased from Zhejiang Luda Mechanical Instrument Co., Ltd. (Shaoxing, China), with a maximum test force of 2000 kN (Figure 4). This machine was loaded by controlling the force. The loading rates for the cube compressive strength tests were 5–8 kN/s and 0.5–0.8 kN/s for bending and splitting tensile strength tests. The failure loads of the specimens were recorded, and the mean of three specimens was determined as the test value. Nonstandard specimens of 100 mm × 100 mm × 100 mm were selected for cube compressive and splitting tensile strength tests and 100 mm × 100 mm × 400 mm for flexural strength tests. The strength values are calculated as follows [49]:

(1) $$\mathrm{Compressive}\;\mathrm{strength}\;f_{c}=0.95\times \frac{F}{A}$$

(2) $$\mathrm{Splitting}\;\mathrm{tensile}\;\mathrm{strength}\;f_{sp}=0.85\times \frac{2F}{\pi A}$$

(3) $$\mathrm{Flexural}\;\mathrm{Strength}\;f_{f}=\frac{Fl}{bh^{2}}$$

where 0.95 and 0.85 are the size conversion coefficients when converting nonstandard test pieces into standard ones; $f_{c}$ and $f_{sp}$ present the compressive and splitting tensile strengths of the specimens; F is the ultimate load on the loading curve; and b, h and A denote the width, height and area of the specimens, respectively.

The porosity and micropore size distribution of DSC samples were analyzed using MIP tests. After standard curing for 28 d, a sample with a 10 mm diameter was selected from the specimen core and soaked in isopropyl anhydrous ethyl alcohol for 24 h to stop hydration. Before the tests, the samples were dried to a constant weight in an oven at 60 °C for 48 h [51]. Then, the tests were performed on a Poremaster33 automatic mercury injection aperture analyzer. The aperture ranged from 0.003 to 1000 µm, and the pressure was 30,000 Pa (Figure 5). 

Before the tests, the cube specimen (100 mm^3^) was broken to obtain a sample with a diameter of about 10 mm, and the obtained sample was washed and dried naturally. Then, it was ground and pasted on a conductive film and gold-sprayed in Cressington 108 coater (Cressington scientific instruments Ltd., Watford, UK) at 40 s injection time and 30 mA current to ensure electrical conductivity. SEM images at different magnifications were obtained using a JSM-7610F mode field emission scanning electron microscope (Japan Electronics Co., Ltd., Tokyo, Japan) (Figure 5).

## 3. Results and Discussion

### 3.1. Workability

**Figure 6:**
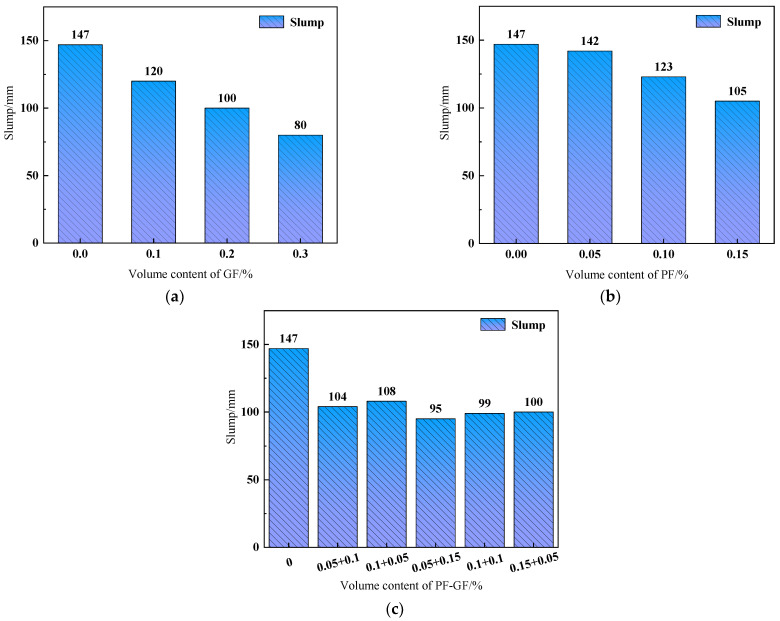
Slump of FRDSC mixtures: (**a**) GFRDSC; (**b**) PFRDSC; (**c**) HyFRDSC.

Slump is an important indicator of concrete workability [52]. The slump test results of DSC are summarized in Figure 4, where the slump decreases with the increase in fiber content. Consistent results were reported in the previous literature. For example, SINGH M et al. [53] found that excessive fiber incorporation mitigated the workability of the concrete mix and affected fiber dispersion. It can be seen from Figure 6 that the slump of DSC decreases by 45.6%, 28.6% and 35.4% at the maximum GF, PF and hybrid-fiber contents of 0.3%, 0.15% and 0.2%, respectively, indicating that the fiber content significantly affects the fluidity of DSC. The main reason is that the cement mortar wraps around the fiber surface, and a higher fiber content requires more mortar on the surface, leaving less mortar to encapsulate the aggregate. Consequently, the physical friction between the aggregate and the mortar is enhanced, and the DSC fluidity is reduced, i.e., the slump. A comparison between Figure 6a,b suggests that the slump of GFRDSC is slightly smaller than that of PFRDSC under the same fiber content. As shown in Figure 6c, the slump of HyFRDSC decreases with the upgrading GF content. With 0.2% fiber content and the PF-to-GF ratio of 1:3, the slump of the reinforced DSC is 35.4% lower than that of the reference DSC. The main reasons are as follows: (1) With a higher water absorption rate than PF, GF surface adsorbs less free water, leading to lower DSC fluidity. (2) Under the same weight, GFs with a smaller diameter than PFs have a greater specific surface area, which requires more cement slurry to wrap around the surfaces and reduces the free cement slurry, intensifying DSC slump reduction [54].

### 3.2. Failure Process

**Figure 7:**
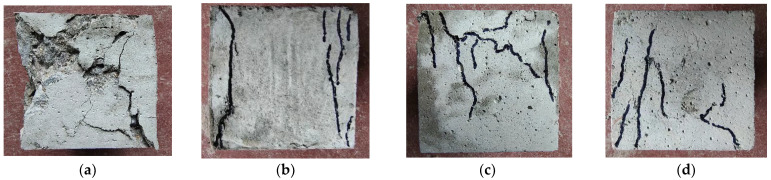
Compressive failure morphologies. (**a**) Reference DSC; (**b**) PFRDSC; (**c**) GFRDSC; (**d**) HyFRDSC.

**Figure 8:**
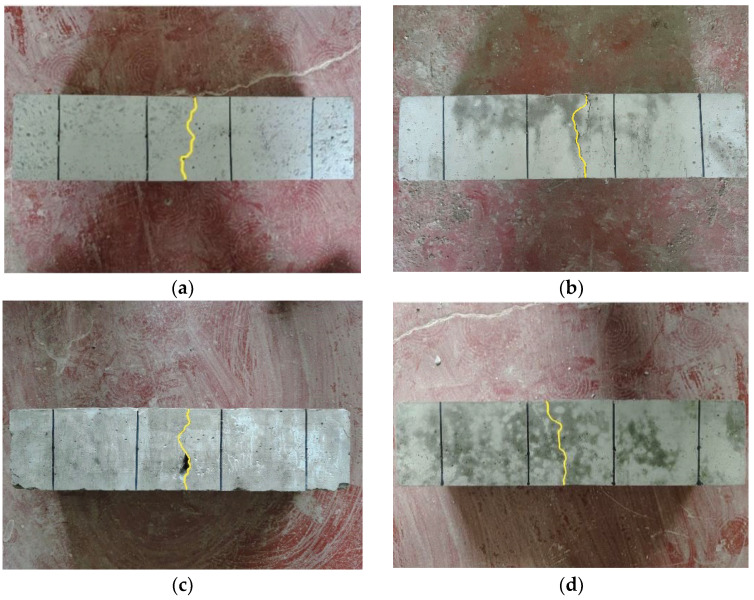
Flexural failure morphologies. (**a**) Reference DSC; (**b**) PFRDSC; (**c**) GFRDSC; (**d**) HyFRDSC.

**Figure 9:**
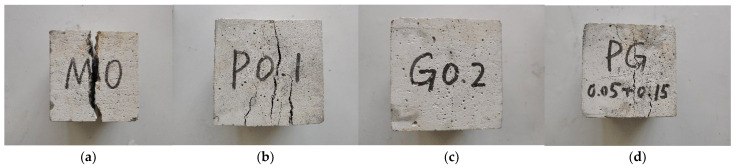
Splitting tensile failure morphologies. (**a**) Reference DSC; (**b**) PFRDSC; (**c**) GFRDSC; (**d**) HyFRDSC.

The failure morphologies of each specimen are shown in Figure 7, Figure 8 and Figure 9. In Figure 7a, Figure 8a and Figure 9a, initial cracks immediately occur at the corner of the reference DSC specimen under stresses and gradually penetrate through the specimen; these cracks continue to expand in range and width, resulting in large spalling areas around the specimen. The DSC specimens under tensile and bending loads suddenly break into two parts when the loads reach the bearing capacity, and obvious brittle failure characteristics are observed.

After adding fibers, the damage characteristics of the specimen changed greatly. As shown in Figure 7b–d, the original cracks mostly appear at the corners under compression loading and expand longitudinally, and the “sizzle” sound of fiber fracture can be heard. The crack range and width of the specimen are smaller than those of the reference DSC, and only a few fragments are spalling, indicating good integrity. When the ultimate bearing capacity is reached, the specimen does not break suddenly and shows certain ductility instead. The loading speed of the press machine slowly drops to zero.

Figure 8b–d demonstrate that microcracks appear on the surface of DSC specimens considering the flexural strength, accompanied by fiber fracture sounds weaker than those upon breakage. When the specimen sustains damage under continuous loading, crack expansion and penetration occur, and the specimen breaks into two parts with a large splitting sound. Additionally, the loading speed decreases to zero, and the specimen shows obvious plastic failure characteristics.

It can be seen from Figure 9b–d that the splitting tensile damage morphologies of mono- and hybrid-FRDSC specimens are consistent. With the increasing load, microcracks emerge in the middle of the specimen with the sound of fibers pulling out and breaking. Upon the ultimate bearing capacity, these small cracks evolve into multiple macrocracks and fail to penetrate the specimen, resulting in relatively complete damage without obvious fragmentation. Similarly, the loading speed slowly drops to zero, and the specimen exhibits plastic failure characteristics.

### 3.3. Strength Analysis

#### 3.3.1. Compressive Strength

Figure 8 shows that compared with the reference DSC, the compressive strength of FRDSC decreases first and increases before decreasing again with the increasing fiber content. DSC specimens with 0.05% PFs or 0.1% GFs exhibit reduced compressive strength by 1.4% and 3.1%, respectively. In contrast, those with more PFs (0.1% and 0.15%) or GFs (0.2% and 0.3%) display an increased compressive strength (9.6% and 6.2% vs. 6.7% and 0.5%). According to the above analysis, an appropriate fiber content has the optimal enhancing effect on DSC. Excessively low or high fiber contents adversely affect the compressive strength of DSC; the former is insufficient for the fiber to fully play a leading role in the matrix crack expansion, and the latter leads to poor fiber dispersion and agglomeration. The weak layers in the interfacial transition zone of DSC are increased, and the compressive strength decreases. LIN et al. [25] reported that that the compressive strength of some PP-PVA-reinforced concrete was lower than those of PVA-fiber-reinforced concrete alone due to excessive fiber admixture.

**Figure 10:**
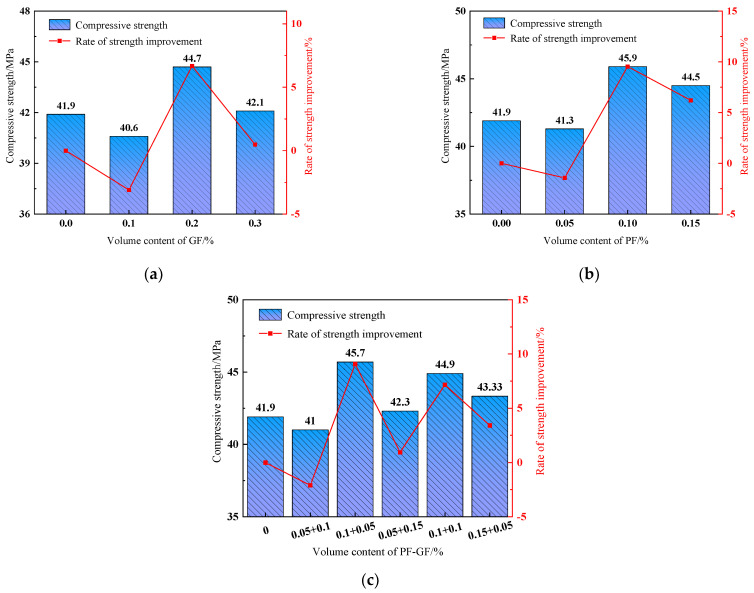
Compressive strength. (**a**) GFRDSC; (**b**) PFRDSC; (**c**) HyFRDSC.

Figure 10a,b suggest that PFs have a significantly better effect on DSC compressive strength than GFs. As shown in Figure 8c, the compressive strength of HyFRDSC shows an increasing trend overall. With a hybrid-fiber content of 0.15% and a 2:1 PF-to-GF ratio, the compressive strength of DSC maximizes and surpasses that of the reference one by 9.1%. With a hybrid-fiber content of 0.2% and a PF-to-GF ratio of 1:1, the compressive strength of DSC is increased by 7.2%. Moreover, the positive effect of the hybrid fiber is fully achieved, and the crack development is restricted.

#### 3.3.2. Flexural Strength

**Figure 11:**
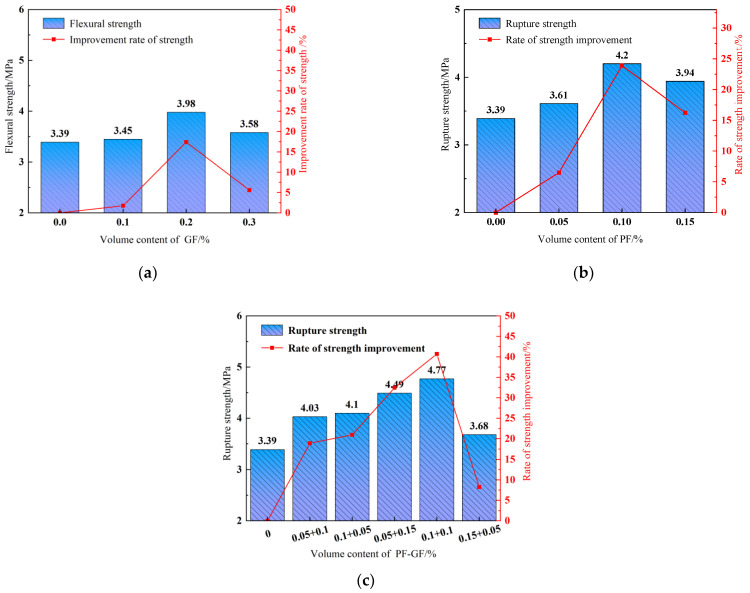
Flexural strength. (**a**) GFRDSC; (**b**) PFRDSC; (**c**) HyFRDSC.

As shown in Figure 11, the flexural strength of the reference DSC is 3.39 MPa, and the fibers considerably improve the flexural strength, which increases first and then decreases with the elevating fiber content. For 0.1% PFs or 0.2% GFs, the DSC flexural strength increases by 23.9% or 17.4%, respectively. The reason is that the tightly bonded fiber and the cement-based composite material bear part of the stresses generated by the DSC shrinkage and deformation during loading with the appropriately increasing fiber content, inhibiting the development of small cracks and improving the flexural strength. The enhancing effect of PFs on DSC flexural strength is slightly greater than that of GFs, as illustrated in Figure 11a,b. Figure 11c showcases that the hybrid fibers noticeably enhance flexural strength. With a hybrid-fiber content of 0.2% and a PF-to-GF ratio of 1:1, the DSC flexural strength is increased by 40.7%, achieving an optimal enhancing effect. Furthermore, the effect of the hybrid fibers on the DSC flexural strength is better than that of monofibers.

#### 3.3.3. Splitting Tensile Strength

**Figure 12:**
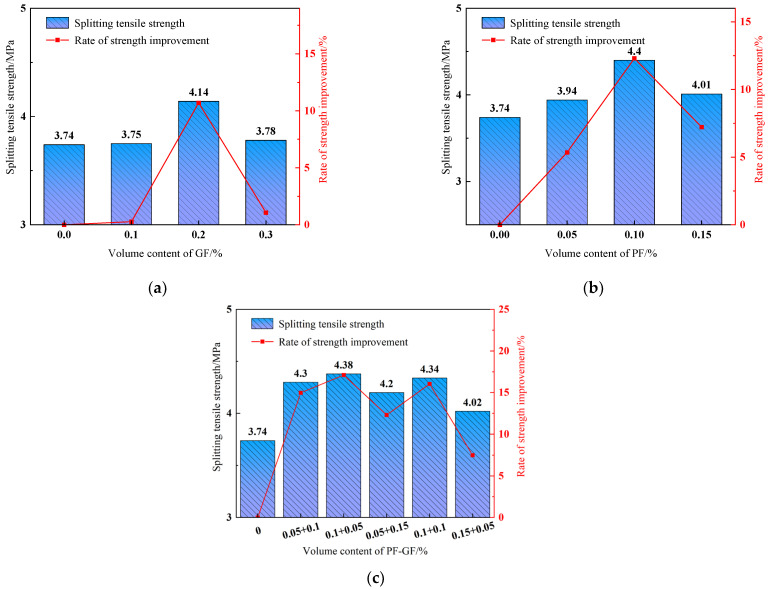
Splitting tensile strength. (**a**) GFRDSC; (**b**) PFRDSC; (**c**) HyFRDSC.

Figure 12 shows that adding fibers dramatically improves the DSC splitting tensile strength. As seen in Figure 12a,b, the DSC splitting tensile strength increases with the addition of GFs or PFs, showing a first increasing and then decreasing trend. The splitting tensile strength increments are the largest in DSC specimens with 0.1% PFs or 0.2% GFs, reaching 17.65% and 10.70%, respectively. This is attributed to the fact that the evenly dispersed fibers in the matrix play a bridging role in transferring loads, relieving the concentrated stress at the crack edge and maintaining the uniform and continuous matrix stress. Consequently, the DSC splitting tensile strength is improved. With the increase in fiber content, the weak transition zone at the DSC interface expands, resulting in stress concentration at the crack and splitting tensile strength deterioration. Research [40] found that the splitting tensile strength of fiber-reinforced concrete containing 1.35% PF was 72.8% lower than that of plain concrete, which was mainly due to the high-fiber admixture. Figure 12c shows that the hybrid fibers significantly enhance the DSC splitting tensile strength. In the presence of 0.15% hybrid-fiber content and 2:1 PF-to-GF ratio, the splitting tensile strength increases by 17.11%, exhibiting an optimal enhancing effect.

In conclusion, the hybrid fibers with different contents and mixing ratios significantly improve the DSC splitting tensile strength and outperform the monofibers. For one thing, a higher PF content has a “bleed air” effect, which increases the small and stable bubbles in the specimen and inhibits the development of early primary cracks. For another, the distributed GF bears the tensile stress of the matrix, hindering the development of macrocracks in the later stage. This combination can effectively improve the DSC splitting tensile strength.

#### 3.3.4. Flexural/Compressive and Tensile/Compressive Strength Ratios

- Flexural/compressive strength ratio.

**Figure 13:**
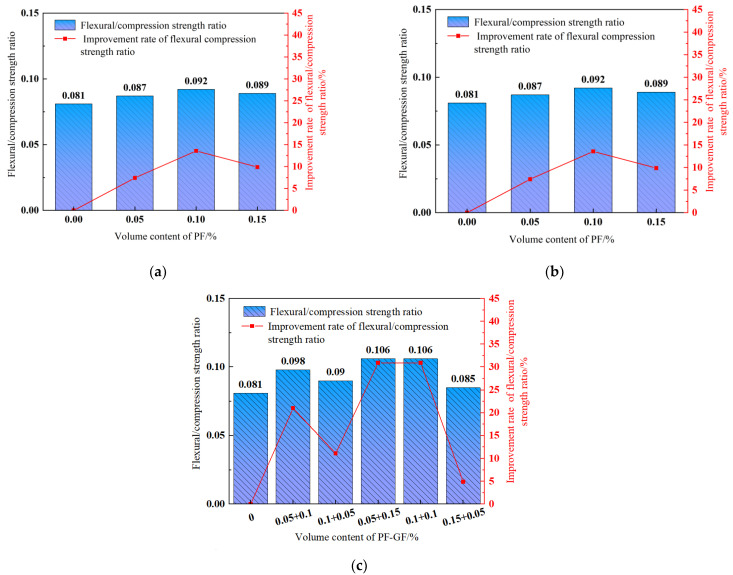
Flexural/compressive strength ratio. (**a**) GFRDSC; (**b**) PFRDSC; (**c**) HyFRDSC.

The flexural/compressive strength ratio roughly measures the concrete toughness [55], and its variation trend with fiber contents is depicted in Figure 13. The bending pressure of each fiber-reinforced group is significantly higher than that of the reference DSC. In contrast, a higher fiber content exhibits a stronger compressive strength and flexural strength of DSC, and the effect on the flexural strength is significantly greater than that of compressive strength. Akca et al. [34] also found that the effect of PF on the flexural strength of concrete was greater than on the compressive strength.

It is indicated that the flexural/compressive strength ratio is improved in a first increasing and then decreasing trend with the increasing fiber content. Figure 13a,b show that the flexural/compressive strength ratio in 0.2% GF or 0.1% PF content maximizes at 9.88%, 13.58% higher than the reference DSC. Figure 11c demonstrates that with the hybrid-fiber content of 0.2% and the GF-to-PF ratios of 1:3 and 1:1, the flexural/compressive strength ratio maximizes, reaching an increase of 30.86% in comparison to the reference DSC. In sum, adding fibers significantly improves the DSC flexural strength, and the compressive strength improvement remains relatively stable, suggesting an improved flexural/compressive strength ratio and toughness.

- Tensile/compressive strength ratio.

**Figure 14:**
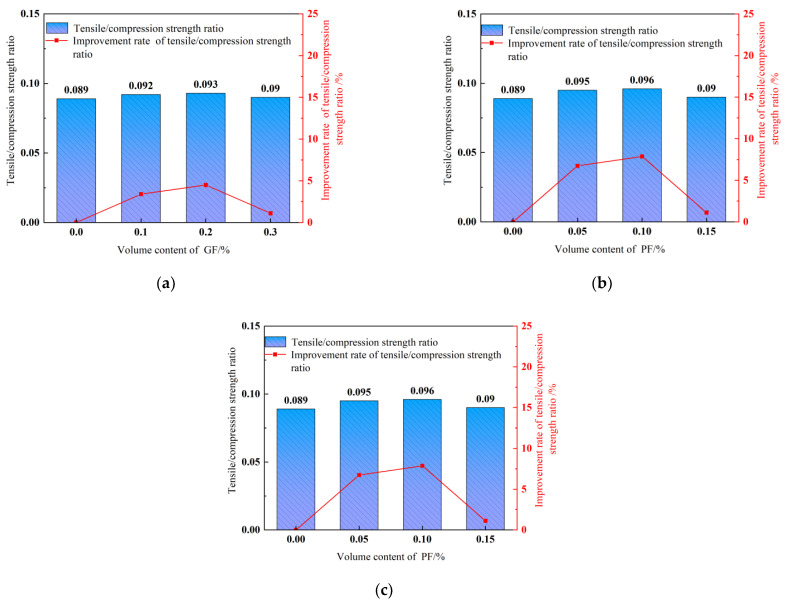
Tensile/compressive strength ratio. (**a**) GFRDSC; (**b**) PFRDSC; (**c**) HyFRDSC.

The tensile/compressive strength ratio assesses the brittleness of concrete. A smaller tensile/compressive strength ratio means greater brittleness and lower toughness [56]. Figure 14 reveals the significant increase in FRDSC tensile/compressive strength ratios. As shown in Figure 14a,b, the growth rates of tensile/compressive strength ratios are the highest in specimens with 0.2% GFs or 0.1% PFs, reaching 9.88% and 13.58%, respectively, suggesting that PFs have a more significant effect on DSC splitting tensile strength than GFs. As shown in Figure 12c, the tensile/compressive strength ratio growth rates in each group with GFs or PFs is above 10% except for the 0.15% GF and 0.05% PF groups, and the highest is 30.86%. In contrast, hybrid fibers deliver a better effect on the tensile/compressive strength ratio than monofibers. The GF-PF hybrid combination improves the compressive and splitting tensile strength of the DSC matrix and effectively overcomes the shortcomings of high brittleness and low toughness.

The variation trends of the flexural/compressive and tensile/compressive strength ratios are insufficient to determine whether a higher fiber content leads to more DSC defects, which reduce the compressive strength and affect the flexural strength and splitting tensile strength. However, the flexural/compressive and tensile/compressive strength ratios are still higher than those of the reference DSC. Mixing a proper quantity of PF and/or GF into DSC improves the concrete toughness.

### 3.4. Pore Structure

Specimen groups P0.1, G0.2, PG0.1 + 0.5 and M0 (control group) were selected for mercury injection capillary pressure tests, and the effects of pore distribution and porosity on the mechanical properties of specimens under the optimal fiber content were studied through microstructure analysis.

**Figure 15:**
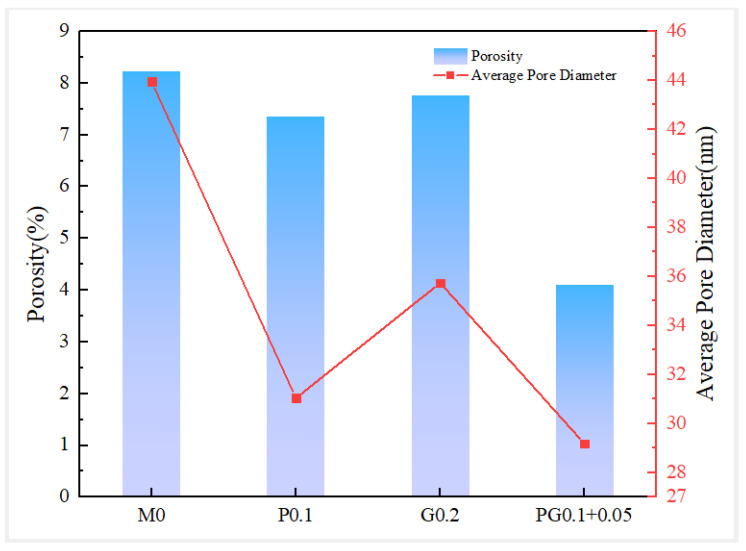
Porosity and average pore diameter of FRDSC.

As shown in Figure 15, the porosity and average pore size are significantly reduced compared to the reference DSC. For instance, the porosity of PFDSC and GFDSC decreases by 10.7% and 5.93%, respectively. The porosity of HyFRDSC drops significantly by 50.01%. The average pore sizes of PFDSC and GFDSC are 31.04 nm and 35.74 nm, respectively, reducing by 29.4% and 18.68% compared with the reference DSC. The average pore size of HyFRDSC is decreased most markedly by 33.61%. It is indicated that hybrid fibers have the best effect mainly due to the addition of hydrophilic GFs increasing the water–binder ratio. In so doing, the binding strength of GFs and cement substrate is strong, significantly reducing the porosity. PFs have good dispersion and small fiber spacing, allowing increased DSC density and reduced porosity. A reasonable hybrid-fiber proportion can give full play to the positive hybrid effect, effectively refine the pore structure, improve the density and reduce the porosity and average pore size.

**Figure 16:**
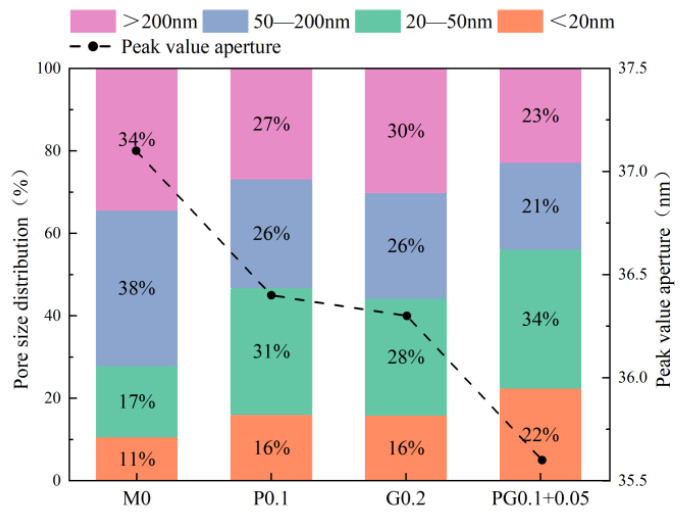
Pore size distribution and peak pore diameter.

Figure 16 shows the pore size distribution and peak pore size (all below 50 nm) of the specimens. It can be seen from Figure 16 that the pores with pore structure greater than 200 nm account for 34.36% of the total pore volume. Compared with the reference DSC, the pore volume ratio in the small pore segment below 20 nm and 20–50 nm is significantly increased in the DSC mixed with fiber, while the pore volume ratio in the harmful pore segment is correspondingly decreased in the large pore segment. Hybrid fibers can increase the volume ratio of pores below 20 nm to 22.46% and reduce the volume ratio of pores above 200 nm to 22.77%. In this sense, fiber reinforcement can improve the internal pores of DSC, effectively limit the formation of harmful pores and reduce the conversion of harmful pores to harmless and less harmful ones [39,40]. The pore size distribution of HyFRDSC is better than that of monofiber-reinforced DSC, indicating that a reasonable hybrid-fiber content can fully reduce the large pores in the DSC matrix and improve the internal compactness.

### 3.5. Micromorphological Analysis

#### 3.5.1. Cement Slurry Microstructure

**Figure 17:**
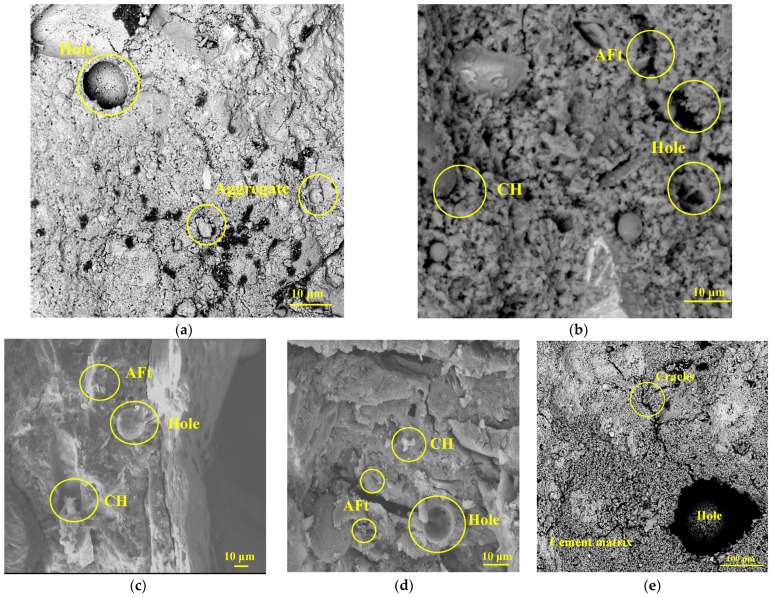
SEM images of FRDSC: (**a**) reference DSC; (**b**) hydration product of the reference DSC; (**c**)> GFRDSC; (**d**) PFRDSC; (**e**) HyFRDSC.

The cement slurry microstructure is presented in Figure 15. The microstructure of the reference DSC is significantly different from that of the fiber-reinforced DSC. As shown in Figure 17a, the internal structure of the reference DSC under magnification of 300 times is loose, and the bond between cement and fine aggregate is not tight enough, with obvious pores and microcracks. Figure 17b shows the micromorphology of the hydration products in the reference DSC. Calcium silicate hydrate (C-S-H), Ca(OH)_2_ crystal (CH) and aft-ettringite (AFt) generated during hydration show flocculent and honeycomb structures under 4000× magnification. A large number of C-S-H gels bind and cling, covering and wrapping crystals (i.e., CH). In addition, the independent aggregate, cement and other hydration products in the matrix are tightly bonded and form a spatial grid structure to maintain the DSC strength. However, the original dense stress structures of some thin interfacial DSC cause macromechanical properties to deteriorate [51,52,53].

According to Figure 17c–e, the fibers improve the overall microstructure density and uniformity of the cement. The FRDSC surface is smoother and denser than that of the reference DSC. Due to the good hydration reaction of the gelled material and the filling and plugging of various hydrates, the sizes of pores and microcracks in the matrix are obviously reduced, consistent with the phenomenon found in the literature. This is similar to the phenomenon found in the microscopic study of steel-fiber–PF-reinforced concrete in the literature [19].

Both PF and GF specimens contain substantial C-S-H gels, of which the joints are joined and set together. In this case, hydration products and solute particles are tightly packed with C-S-H gels to form a dense and continuous phase. As illustrated in Figure 17e, the HyFRDSC microstructure is the densest and most uniform of all specimens. The C-S-H gels are also well developed, and the solidified gels exhibit a closed bonding surface and embed with each other, granting the cement slurry strong bonding properties.

#### 3.5.2. Fiber Microstructure and Toughening Mechanism

**Figure 18:**
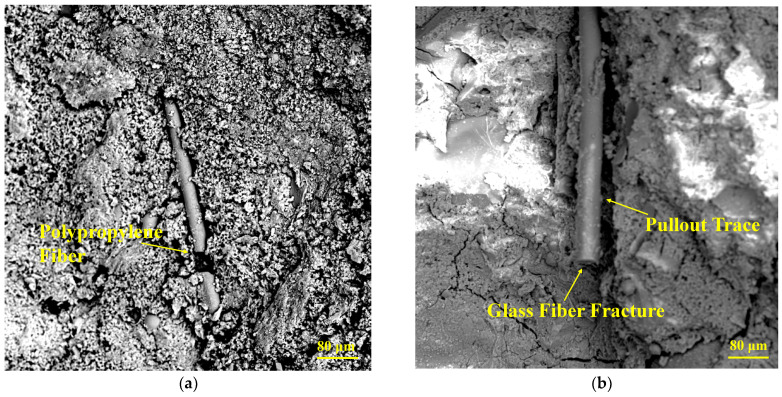
Fiber micromorphology. (**a**) PFs; (**b**) GFs.

Figure 16 shows the micromorphology of fibers. In Figure 18a, the uniformly and randomly distributed PF filaments adhere to the DSC matrix through the “bridging” effect, which improves the structural integrity and strength. In addition, PFs break due to yielding with the increasing stress. As shown in Figure 18b, GFs exhibit a fracture failure mode with obvious pull-out traces, which are induced by the phenomenon that the internal stress in GFs exposed to early loading is greater than the interfacial force between the fiber and DSC and smaller than the fiber yield load. When the load increases, the internal stress is greater than the yield load of GFs, leading to fracture and failure [39].

Under the loading conditions, internal stresses in different directions appear in the DCS matrix. The reference DSC matrix bears the external load alone, and the cracks develop from the inside to the surface with the progressive loading, breaking the specimen. The resistance stress of FRDSC mainly includes the interfacial adhesion between the matrix and the fiber, the tensile strength of the fibers, the friction force between the aggregates and the bearing capacity of the matrix. With the increasing internal stress, independent and incomplete microcracks appear in the FRDSC matrix. Since the elastic modulus of the fiber is greater than that of the DSC matrix, internal stress tends to appear in the microcracks, which develop rapidly. Figure 18a shows that when these microcracks expand to the fiber, the fiber exerts its “bridging” effect. If the internal stress is below the PF yield load, it absorbs and transmits the remaining stress and fractures otherwise. At this point, the energy released from the PF broken end is dispersed in a “ring” manner to the matrix and the surrounding fiber. The random distribution of PFs in the matrix can effectively prevent the generation and development of microcracks, thus improving the bearing capacity. When the load increases further, macrocracks appear on the surface. GFs can cross the cracks and restrain crack development until breakage due to high elastic modulus and tensile strength, effectively hindering the formation and development of macrocracks [51,55]. In this sense, the positive effect of hybrid fibers can be fully utilized under appropriate fiber contents and mixing ratios, greatly improving the flexural strength of DSC.

## 4. Conclusions

(1) The slump of FRDSC with different fibers decreases with the increasing fiber content. The slump of HyRDSC decreases with the increase in GF volume. When the mixture amount is 0.2% and the mixture ratio of PF to GF is 1:3, the slump of HyFRDSC decreases by 35.4%.
(2) FRDSC shows obvious plastic characteristics upon compressive, flexural and splitting tensile fractures. The strength improvements from fiber reinforcement rank as flexural strength > splitting tensile strength > cube compressive strength. The optimum PF and GF contents are 0.1% and 0.2%, respectively. The effect of PFs on the DSC compressive and tensile strengths is better than that of GFs. In the case of the hybrid fiber of 0.1% PF + 0.1% GF, the DSC flexural strength is increased by 40.7%. For 0.1% PF + 0.05% GF content, the compressive strength and splitting tensile strength are enhanced by 9.1% and 17.11%, respectively.
(3) Compared with the reference DSC, the flexural/compressive strength ratios of GFRDSC, PFRDSC and HyFRDSC are increased by 9.88%, 13.58% and 30.86%, respectively; their tensile/compressive strength ratios are increased by 4.5%, 5% and 18%, respectively. Fibers enhance DSC toughness, and the improvement effect of the hybrid fiber is relatively significant.
(4) The effect of hybrid fibers on the internal pores of DSC is more significant than that of single fibers. The porosity and average pore size of HyDSC decrease by 50.01% and 33.61%, respectively. In addition, the pore volume ratio below 20 nm increases to 22.46% and that above 200 nm decreases to 22.77%.
(5) HyFRDSC has the most dense and homogeneous microstructure among the mixtures. PFs alleviate the stresses in a “bridging” manner until yielding, and the damage form is dominated by fracture. For GFs, at the initial stress stage, the internal stress is greater than the interface force between the fiber and DSC and less than the yield load of the fiber, so the fiber has obvious drawing marks. When the internal stress is greater than the yield load of the fiber, the fiber will also eventually have fracture damage.

## Figures and Tables

**Table 1:** Cement parameters and chemical compositions.

Ignition Loss/%	Specific Surface Area m^2^/kg	Initial Setting Duration/min	Final Setting Duration/min	Stability	Flexural Strength /MPa	Compressive Strength /MPa	Cl^−^/%	SO_3_/%	MgO/%
3.49	349	187	246	Qualified	5.8	30.7	0.032	2.41	3.32

Note: The flexural and compressive strengths are both 3D strengths.

**Table 2:** Physical properties of fine aggregates.

Fine Aggregate	Bulk Density	Apparent Density	Fineness Modulus	Mud Content	Superplasticizer
Unit	kg/m^3^	kg/m^3^		%	%
River sand	1558	2578	2.3	2.5	0.8
Desert sand	1542	2602	1.1	1.2	2.1

**Table 3:** Parameters and chemical composition of fly ash.

Ignition Loss/%	Moisture Content/%	Density g/cm^3^	Bulk Density g/cm^3^	AI_2_O_3_/%	AIO_2_/%	Cl^−^/%	SO_3_/%	CaO/%	Fe/%	Free CaO/%
2.8	0.85	2.55	1.12	24.2	45.1	0.015	2.1	5.6	0.85/	0.85

**Table 4:** Performance parameters of PFs and GFs.

Fiber Type	Density/g·cm^−3^	Diameter/μm	Length/mm	Tensile Strength/MPa	Elastic Modulus/GPa	Elongation after Fracture/%
PFs	0.9	31.86	12	567	5.2	39
GFs	2.4		18	2500	70	3.6

**Table 5:** Concrete reference mix ratio/(kg·m^−3^).

Desert Sand	Water	Cement	Fly Ash	Sand	Stone	Water-Reducing Agent
225	181	362	40	524	1333	3.2

**Table 6:** Test groups/(kg·m^−3^).

Specimen No.	PF/(kg·m^−3^)	GF/(kg·m^−3^)
M0	0	0
P0.05	0.45	0
P0.1	0.9	0
P0.15	1.35	0
G0.1	0	2.4
G0.2	0	4.8
G0.3	0	7.2
PG0.05 + 0.1	0.45	2.4
PG0.1 + 0.05	0.9	1.2
PG0.05 + 0.15	0.45	3.6
PG0.1 + 0.1	0.9	2.4
PG0.15 + 0.05	1.35	1.2

Note: P, G and PG indicate PF, GF and PF-GF hybrid fibers, respectively. The subsequent numbers represent the fiber content. For example, PG0.05 + 0.1 refers to a mix of 0.05% PFs and 0.1% GFs, and the reference control group is M0.

## Data Availability

Data are contained within the article.
